# Retraction: Proteomics Approaches for Identification of Tumor Relevant Protein Targets in Pulmonary Squamous Cell Carcinoma by 2D-DIGE-MS

**DOI:** 10.1371/journal.pone.0285077

**Published:** 2023-04-24

**Authors:** The PLOS ONE Editors

Following the publication of this article [1], concerns were raised regarding results reported in multiple figures. Specifically,

- The 2D gel electrophoresis image in Figure 1A in [1] appears similar to Figure 1A in [2], Figure 2A in [3], and Figure 1A in [4].
- In the SAP and β-actin panels presented in Figure 4A, there appear to be vertical discontinuities suggestive of splice lines at the following locations:
  - Between 2T and 3N.
  - Between 13T and 14N.
  - Between 19T and 20N.

The corresponding author stated that all data underlying the results in this article are available, however they were unable to provide unadjusted original western blot images underlying all the results in Figure 4A.

The corresponding author confirmed that the same 2D gel electrophoresis data are reported in four articles [1–4], and each article focuses on a different aspect of the 2D gel results. The Editors remain concerned that a related study [2] published before this article [1] was not cited, and that the reuse of data from this article [1] was not adequately acknowledged in a subsequent publication [4].

The corresponding author confirmed that SAP panels were spliced in Figure 4A, and provided underlying image data which clarified the original location of the bands. Original image data were not available for some of the corresponding β-actin panels, and some underlying image data for SAP panels were not available without adjustments to contrast/brightness. Therefore, the image data provided did not fully resolve the concerns.

Underlying image data provided for APO-A1 and β-actin panels in Figure 4A appear to have discrepancies with the published panels and similarities were identified between images when color levels were adjusted. The Editors remain concerned about the reliability of these results.

In light of the concerns affecting multiple figures that question the reliability of these data, the Editors retract this article.

LH and SS agreed with the retraction. SS stands by the article’s findings. All other authors either did not respond directly or could not be reached.
